# 17β-Estradiol (E2) may be involved in the mode of crustacean female sex hormone (CFSH) action in the blue crab, *Callinectes sapidus*

**DOI:** 10.3389/fendo.2022.962576

**Published:** 2022-07-25

**Authors:** Tao Wang, Ke He, Lee Blaney, J. Sook Chung

**Affiliations:** ^1^ Department of Marine Biotechnology & Institute of Marine and Environmental Technology, University of Maryland Baltimore County, Baltimore, MD, United States; ^2^ Institute of Marine and Environmental Technology, University of Maryland Center for Environmental Science, Baltimore, MD, United States; ^3^ Department of Chemical, Biochemical, and Environmental Engineering, University of Maryland Baltimore County, Baltimore, MD, United States

**Keywords:** 17β-estradiol (E2), crustacean female sex hormone (CFSH), steroidogenesis, sexual differentiation, *Callinectes sapidus*

## Abstract

17β-estradiol (E2) has been proved to control reproduction, sexual differentiation, and the development of the secondary sexual characteristics of vertebrate females. In decapod crustacean species, crustacean female sex hormone (CFSH), a protein hormone, is required for developing adult-specific ovigerous setae for embryo brooding and gonophores for mating at the blue crab *Callinectes sapidus* puberty molting. However, it is unclear that whether the mode of CFSH action involves a vertebrate-type sex steroid hormone in crustaceans. To this end, E2 levels were first measured using a competitive ELISA in the hemolymph and the potential CFSH target tissues from both prepuberty and adult females; the presence of E2 was further confirmed with a liquid chromatography tandem mass spectrometry method. Then, the cDNAs of the following genes known to be associated with vertebrate steroidogenic pathways were isolated: StAR-related lipid transfer protein 3 (*StAR3*); 3β-hydroxysteroid dehydrogenase (*3βHSD*); two isoforms of 17β-hydroxysteroid dehydrogenase 8 (*17βHSD8*); and, estradiol-related receptor (*ERR*). RT-PCR analysis revealed that these genes were widely distributed in the eyestalk ganglia, hepatopancreas, brain, ovary, spermathecae, ovigerous and plumose setae tissues of adult females. The *17βHSD8* transcripts were localized in the follicle cells, the periphery of the nuclear membrane of primary oocytes, and yolk granules of the vitellogenic oocytes using *in situ* hybridization, and the corresponding protein was detected in the follicle cells and ooplasm of primary oocytes using immunohistochemistry. Furthermore, the adult females injected with CFSH-dsRNA (*n* = 30 times) had E2 and *StAR3* transcripts levels lower in the ovigerous and plumose setae, spermathecae than controls. These results suggested that the mode of CFSH action in *C. sapidus* might involve E2 in these adult-female-specific tissues.

## Introduction

17β-estradiol (E2) is a female-dominant sex steroid hormone in vertebrates that exerts critical functions in reproduction and sexual differentiation (1, 2). The presence of E2 has been documented in various crustaceans, including the Norway lobster *Nephrops norvegicus* (3), the black tiger prawn *Penaeus monodon* (4), the kuruma prawn *Marsupenaeus japonicus* (5), the mud crab *Scylla paramamosain* (6) and the Chinese mitten crab *Eriocheir sinensis* (7), where E2 functions are implicated in reproduction including vitellogenesis (8, 9), oocyte development and ovary maturation (4, 10, 11). Moreover, E2 induces feminization in amphipods, such as *Gammarus duebeni celticus*, *G. pole*, and *G. pseudolimnaeus* (12). However, the evidence for *de novo* synthesis of E2 in crustaceans needs to be further confirmed as E2 is taken up *via* the diet because many plants contain vertebrate-like sex steroids (13).

On the other hand, the cytochrome P450 (CYP) proteins, hydroxysteroid dehydrogenase (HSD) enzymes, and sex steroid receptors known to be involved in the vertebrate steroidogenic pathway (14, 15) were identified from transcriptomic analyses of several crustaceans. For example, *3βHSD* and *17βHSD* transcripts were found in the gonad tissues of *Portunus trituberculatus* (16) and the patopancreas, ovary, and central nervous systems of *Macrobrachium rosenbergii* together with steroidogenic acute regulatory protein (*StAR*)-related lipid transfer protein and estradiol receptor (*ER*) (17). In *M. japonicas*, the enzyme activity of *17βHSD* and 17α-hydroxylase/C-20–22-lyase (P450c17/Cyp17a) was detected in the ovary, and *3βHSD* and 17α-hydroxylase activities were identified in the hepatopancreas (18). These findings indicate the presence of a vertebrate-like steroidogenic pathway and possibly endogenous sex steroids in crustaceans.

Decapod crustaceans are in general sexually dimorphic in that adult-related features develop at the puberty molt. Crustacean female sex hormone (CFSH), identified from the eyestalk of the female blue crab *Callinectes sapidus*, regulates developing adult-female-specific reproductive phenotypes of ovigerous setae for embryo brooding and gonopores for mating at the puberty-terminal molting (19). Like *P. pelagicus* (20) and *P. spinimanus* (21), *C. sapidus* females tend to produce several spawns during their adulthood (22) and re-utilize the brooding ovigerous setae for each spawning. However, the action mode of CFSH is unknown in these adult-female-specific tissues. Considering the prominent functions of E2 in female reproduction and sexual differentiation, we proposed that the action mode of CFSH may involve E2 in the adult-female-specific tissues that are required for developing brooding and maternal care.

Herein, E2 levels were measured in the hemolymph and potential/putative CFSH target tissues using a competitive ELISA, and the presence of E2 was further confirmed with a liquid chromatography tandem mass spectrometry (LC-MS/MS) method. Subsequently, several steroidogenesis genes, including *StAR3* (StAR-related lipid transfer protein 3), *3βHSD*, two isoforms of *17βHSD8*, and *ERR* (estradiol-related receptor) that were initially identified from the blue crab *C. sapidus* transcriptomes, were analyzed for the tissue distributions by RT-PCR and localized *17βHSD8* mRNAs and its protein in the ovary by *in situ* hybridization and immunohistochemistry, respectively. Moreover, the effects of CFSH on E2 and steroidogenesis gene transcript levels were determined using CFSH-dsRNA injection.

## Materials and methods

### Animal culture

Animals were reared in the blue crab hatchery, Aquaculture Research Center [Institute of Marine and Environmental Technology (IMET), Baltimore, MD]. After reaching prepuberty, the females were monitored for their molt stages, early premolt and late premolt (23). For the mating study, an individual prepuberty female at the E_0_ stage (24) was placed into a 30-liter container, holding 10 liters of 20 ppt artificial seawater (ASW), with an adult male to allow for mating immediately after puberty molting. After mating, adult females with carapace widths of 132.4 ± 3.5 mm (*n* = 10) were kept in five tanks in a closed circulating aquaculture system (91 cm W × 112 cm H × 58 cm D). Each tank held 300 liters of ASW under the following conditions: 16 hours dark, 8 hours light at 18-20°C (22). The water quality was monitored daily by ZooQuatic Lab (Baltimore, MD). Animals were fed about 10% of their body weight with a piece of frozen squid, shrimp, or mussels daily at 8–9 A.M. during the experimental period (Oct. 2019-Feb. 2020).

### Tissue collection

The experimental and prepuberty animals were sacrificed in the afternoon (2–6 P.M.). A hemolymph sample (200 µl) was withdrawn directly into an insulin syringe containing crab anticoagulant solution (*i.e.*, 450 mM NaCl, 10 mM KCl, 10 mM NaHCO_3_, 1 mM EDTA, 10 mM HEPES, pH 7.3) in a 1:1 ratio (v:v). The animals were then placed on ice for 10 min before dissection. The eyestalk ganglia, brain, and ~100 mg of the hepatopancreas, ovary, spermatheca, ovigerous setae, and plumose setae tissues were dissected in ice-cold DEPC-treated crustacean saline (19) under a stereomicroscope (Leica). After two rinses in fresh saline, the tissues were briefly blotted with Kimwipes, collected into a 1.5-ml tube, and placed on dry ice. The wet weights of tissues were measured, and then the tissues were stored at -80°C until further analysis.

### Steroid extraction

The steroid extraction was carried out by following the procedure described in Chao etal. (25). In brief, a single eyestalk ganglion, brain, or 10 mg (wet weight) tissue samples were homogenized in 135 µl ice-cold phosphate buffer saline (PBS). The steroid was extracted by adding 635 µl diethylether to the homogenized sample. The tubes were vigorously vortexed for 1 min, and then centrifuged at 14000 rpm for 10 min at room temperature. The tubes were gently placed in an ethanol/dry ice bath to freeze the aqueous phase, and the lipid phase was transferred to a 4-ml glass tube (Fisher Scientific). The extraction steps were repeated four times with the aqueous phase of each sample. The pooled lipid phase was either dried by air or with a SpeedVac (Jouan). Samples were kept at -20°C for E2 measurement using ELISA or LC-MS/MS.

### E2 measurement

E2 levels in the tissues were estimated using an ELISA kit by following the manufacturer’s protocol (Enzo Biochem). The standard curve was generated using E2 solutions with known concentrations of 29.3 – 30,000 pg/ml. The standards and experimental samples were assayed in triplicate, and the plate was read at an absorbance of 405 nm using a SpectraMax M5 reader (Molecular Devices). The EC_50_ values of ELISAs were 534 ± 12 pg/ml (*n* = 6). Data were presented as follows: mean ± SE pg/tissue (*n* = 5) for the eyestalk ganglia and brain; mean ± SE pg/10 mg wet weight (*n* = 5) of the hepatopancreas, ovary, spermathecae, ovigerous setae, and plumose setae tissues; and, mean ± SE pg/10 µl (*n* = 5) of hemolymph.

### Liquid chromatography with tandem mass spectrometry analysis

The presence of E2 in the brains (adult females) was confirmed by an LC-MS/MS method modified from previous protocols (26–28). First, 100 ng (100 µl × 1 µg/ml) of the E2-d3 surrogate standard (Sigma-Aldrich) was added to the dried lipid phase collected from brain samples (section 2.3) and incubated overnight. After reconstitution in 205 µl PBS, the sample solution was passed through a Sep-Pak^®^ C18 solid-phase extraction (SPE) cartridge (Waters) to remove interfering substances (26). The SPE cartridges were washed with 5 ml of 100% isopropanol and then conditioned with 5 ml MilliQ (Waters) or Hydro water. After loading the 200-µl sample onto the SPE cartridge by gravity, the column was washed with 5 ml of water. Next, E2 and the E2-d3 surrogate standard were eluted from the SPE cartridge with 3 ml of 40% isopropanol, and the first five drops of eluate were removed as void volume. The extracts were dried at 40°C in a vacuum centrifuge (Juan), reconstituted in 220 µl PBS, and diluted to 300 µl with 50% methanol containing 200 ng/ml of the 17-ethinylestradiol-2,4,16,16-d4 (EE2-d4; CDN Isotopes) internal standard.

E2, E2-d3, and EE2-d4 were measured using an UltiMate 3000 LC coupled to a Thermo TSQ Quantum Access Max MS/MS (Thermo). Briefly, 50 µl of the reconstituted SPE extracts were injected onto a Waters XBridge C18 guard column (2.1×10 mm, 3.0 µm) connected to an analytical column (2.1×150 mm, 2.5 µm) with the same material. The mobile phase was comprised of (A) LC-MS grade water with 0.1% NH_4_OH (pH 10.5) and (B) methanol with 0.1% NH_4_OH. Isocratic elution was conducted with 30% A at a flow rate of 200 µl/min. The column compartment was maintained at 40°C, and the overall method run time was 8 min. Negative electrospray ionization mode was employed for LC-MS/MS analysis, and two characteristic product ions were used to quantify and confirm each analyte. The surrogate standard recovery was 89% in the brain sample. The detailed analytical parameters are available in **Table S1** of the supporting information.

### Cloning of the full-length cDNA sequences of StAR3 and 3βHSD

The total RNA (~1-1.5 μg) from the eyestalk ganglia was subjected to 5’ and 3’ rapid amplification of cDNA ends (RACE) cDNA synthesis using the SMART cDNA amplification kit (BD Bioscience) according to the manufacturer’s protocol. Gene-specific primers for 5’ and 3’ RACE (**Table 1**) were designed using NCBI Primer-BLAST based on transcripts pulled from our transcriptomes. The amplified products were purified using a QIAquick gel extraction kit (Qiagen) and ligated to a pGEM-T easy vector (Promega). The recombinant vector was used for transforming *E. coli* competent cells. Clones containing the inserts were isolated and cultured at 37°C overnight for subsequent DNA sequencing (ABI).

**Table 1:** List of primers that were used for 5′ and 3′ RACE, and *in situ* hybridization.

Primers	Purpose	Sequence (5’-3’)
StAR3-1R	5’ RACE	TGAACCGTACAGCAGCAAC
StAR3-2R		ACTGCTGACATACGACCGTT
StAR3-3R		TCCTACCAGAGGAGAGCGGTGGC
StAR3-4R		ATGGCTTTGCAGGTGGTGTA
3βHSD-1R		AGTTGATCACTGCCTCTACGC
3βHSD-2R		ACGATGACTGGATTGGCACA
3βHSD-3R		ATCAATTCCCTGTTGCTCGC
StAR3-1F	3’ RACE	TGGAGTGCCAACGAATGTCA
StAR3-2F		GGAACAAGAGGTTTGCAAAGACA
StAR3-3F		AGTTGGCAGTGATGTAGAACCT
3βHSD-1F		CGGCTGTTGCCTTACTGGAT
3βHSD-2F		TCTTGGATTCGCCAGTCGTC
3βHSD-3F		TACTCACCGCCATACTCGTG
17βHSD8a-ishF	ISH	**TAATACGACTCACTATAGGG**GAAGGGGAAAATCGCCCTGGTTAC
17βHSD8a-ishR		**TAATACGACTCACTATAGGG**CCACCTGTCACTTCCACACTGGCG
17βHSD8b-ishF		**TAATACGACTCACTATAGGG**CTCCCGTCGACTTCCATGGCCAG
17βHSD8b-ishR		**TAATACGACTCACTATAGGG**CATGCCTGATCCTCCTGTGACCT

### Sequence analyses

The open reading frames were identified using ExPasy (https://web.expasy.org). The conserved domain was searched *via* SMART (http://smart.embl-heidelberg.de/). ClustalW (www.genome.ad.jp) was used to align the amino acid sequences, which were then modified by ESPript 3.0 (https://espript.ibcp.fr/ESPript/cgi-bin/ESPript.cgi). The phylogenetic tree was constructed using the neighbor-joining method (MEGA X).

### Tissue distribution in an adult female C. sapidus

Total RNA was extracted from the tissues using QIAzol Reagent (Qiagen) following the manufacturer’s procedures. RNA concentrations were measured using a Nanodrop (Thermo Scientific). Reverse transcription with 1.5 µg total RNA was carried out using a PrimeScrip^RT^ reverse transcriptase reagent kit with gDNA eraser (TaKaRa).

After being diluted with RNase-free water at 12.5 ng/μl, cDNA was amplified for the arginine kinase (AK) gene to examine the quality of each sample (19). The spatial distribution of the steroidogenesis genes, *StAR3*, *3βHSD*, 17*βHSD8a*, 17*βHSD8b*, and *ERR*, was examined in the tissues obtained from an adult female using an endpoint RT-PCR assay with the gene-specific primers reported in **Table 2**, together with *AK* and *eIF4A* (eukaryotic translation initiation factor 4A) as reference genes. The PCR conditions were as follows: an initial denaturation step at 94°C for 2.5 min, followed by 35 cycles of 94°C for 30 s, 57°C for 30 s, and 72°C for 30 s, with a final extension 72°C for 5 min. PCR products were resolved on 1.5% agarose gel and stained with ethidium bromide for visualization.

**Table 2:** Gene specific primers used for RT-PCR and RT-qPCR analyses.

Gene name	PCR type	Sequence (5’ - 3’)	Size (bp)	*r^2^*	Efficiency (%)	Intron insertion	GenBank NO.
StAR3	RT-PCR	Fw: AAGCGGCCAGTAATTGTGAC	872	–	–	Yes	MT013236
		Re: TCATGTCTTACCAACTTGATG					
	qRT-PCR	Fw: CAGGAGACTGACCCACTACT	115	0.98	97.4	Yes	
		Re: CATCAGAAGCGAGAGGAGATTC					
3βHSD	RT-PCR	Fw: CTTCCGCAAAGAGGATCTTG	901	–	–	Yes	MT013238
		Re: CTATAATCTTTTCCAAAAACCTCG					
	qRT-PCR	Fw: CTTCCGCAAAGAGGATCTTG	198	0.99	83.4	Yes	
		Re: AGCTGTGACTCGGACACCTC					
17βHSD8a	RT-PCR	Fw: GTACAAAGCTCCTCCCTGTCT	358	–	–	Yes	MT762163
		Re: TACACCAGCCACCATTGAAGT					
	qRT-PCR	Fw: GTACAAAGCTCCTCCCTGTCT	191	0.99	90.0	Yes	
		Re: AGATGTTGACTATGGCTCCT					
17βHSD8b	RT-PCR	Fw: CTCGTTACAGGTGGCGGTAG	556	–	–	Yes	MT762164
		Re: TTATGCCGGGCAGTACACAG					
	qRT-PCR	Fw: CCTGTTGTGGAAATGGAGGA	108	0.97	91.9	Yes	
		Re: GTCTTCAGCCAGTAGTGCTTTA					
ERR	RT-PCR	Fw: GCCCAACCTTCACCTAAACA	1146	–	–	Yes	MT013240
		Rv: TCACCGCATATGTGATTCTAAC					
	qRT-PCR	Fw: GCCCAACCTTCACCTAAACA	207	0.98	99.9	Yes	
		Re: TTCACAAGATGCCACACCAT					
AK	RT-PCR	Fw: ACCACAAGGGTTTCAAGCAG	398	–	–	Yes	AF233355.1
		Re: CCACACCAGGAAGGTCTTGTT					
	qRT-PCR	Fw: TTCCTCCACCCTGTCCAACC	127	0.97	93.3	Yes	
		Re: GAAGCGGTCACCCTCCTTGA					
eIF4A	RT-PCR	Fw: ACGTCAACATGTCCGACAAA	394	–	–	Yes	DQ667140.1
		Re: TGCGTTTCGTTTGACTTCAC					
	qRT-PCR	Fw: CGGTGGAGACAACAAGGACT	160	0.96	94.5	Yes	
		Re: GGCTGATGGCTTCTCAAAAC					

### Localizations of 17βHSD8 transcripts and protein in the ovary

#### Hematoxylin-eosin staining

Ovaries were dissected and fixed overnight in Bouin’s solution at 4°C. The fixed tissues were progressively dehydrated in ascending ethanol concentrations. The dehydrated tissues were cleared in xylene and embedded in Paraffin wax (60°C). The wax blocks were cut 6 μm in thickness and mounted on APTES (3-aminopropyl triethoxysilane)-coated slides. After being deparaffinized and rehydrated using xylene and a graded series of DEPC-treated ethanol dilutions, respectively, the sections were visualized by staining with hematoxylin and eosin.

#### *In situ* hybridization

An *in situ* hybridization was carried out using digoxigenin (DIG)-labeled sense and anti-sense probes synthesized following T7-mediated *in vitro* transcription using TranscriptAid T7 high yield transcription kit (Roche) from the clone of cDNAs fragment of target genes. With some modifications, *ISH* procedures followed previously described protocols (29). Briefly, the deparaffinized sections were rehydrated stepwise into PBS, and treated with proteinase K (20 μg/ml) for 20 min at 37°C. Samples were incubated for 2 h at 58°C in prehybridization solution (*i.e.*, 50% formamide, 5× SSC (pH 7.0 Quality Biological), 50 μg/ml denatured yeast DNA). Sense or anti-sense probe concentrations were 1 ng/μl, and hybridizations were performed at 58°C overnight. Unbound probes were removed by rinsing with a series of low-salt solutions (*e.g.*, 2× SSC, 0.5× SSC, 0.1× SSC). The presence of mRNAs was localized with a DIG nucleic acid detection kit (Roche). Specimens were photographed using an Olympus micro/DP70 camera mounted on an Echo Revolve microscope (Echo).

#### Immunohistochemistry

Ovary sections were deparaffinized, rehydrated, and rinsed in DEPC-treated, double-distilled water before being washed in PTX (*i.e.*, 50 mM phosphate buffer, 0.05% NaN_3_, 0.5% Triton X-100) 3-5 times for 30-60 min/wash at 20°C. The tissues were blocked in 10% normal lamb serum and incubated with anti-antigen serum diluted 1:1000 for 3 days at 4°C (pre-immune and pre-absorption serum for the negative control), followed by incubation with FITC-conjugated goat anti-rabbit IgG (Thermo scientific) at 1:5000 for 2 days. Washing was repeated in PTX 3-5 times, and samples were kept in 50% glycerol and 50% PTX in the dark until photographic analysis with a Leica SP8 confocal microscope.

### Quantitative real-time PCR

Each cDNA sample containing 25 ng total RNA was assayed in duplicate using Fast SYBR Green Master Mix (Applied Biosystems) and gene-specific primers (**Table 2**) by following the manufacturer’s protocol. Gene-specific standards were produced as described in Chung etal. (30). The coefficients of determination (R^2^) and the average amplification efficiency of samples are listed in **Table 2**. The expression levels of each gene were normalized by the *AK* and *eIF4A* levels in the same cDNA samples (29). Data were presented as mean ± SE (*n* = 5) transcripts/μg total RNA.

### Long-term knockdown study *in vivo*

CFSH-dsRNA preparation and injection were carried out as described (19). The mated adult females were injected twice a week with 10 μg CFSH-dsRNA in 100 μl crustacean saline (*n* = 5) or saline-only controls (*n* = 5). These injection regimes were continued 30 times over four months (Oct. 2019-Feb. 2020). Tissues were sampled (section 2.1) for E2 measurement and expression analysis using RT-qPCR assays.

### Statistical analysis

All data were analyzed using SPSS 25.0 for statistical significance. The data were analyzed in two ways. First, one-way ANOVA was employed with multiple samples (*e.g.*, different tissues), and a non-parametric Kruskal-Wallis test followed by Dunn’s *post-hoc* test was used for multiple group comparisons. Second, the student’s *t*-test was used when two groups (*e.g.*, control and test, or the tissue at different stages) were compared. Significance at *p* < 0.05 was accepted and noted by letters or ‘*’ symbols.

## Results

### E2 levels in the tissues of prepuberty females using ELISA

**Figure 1:**
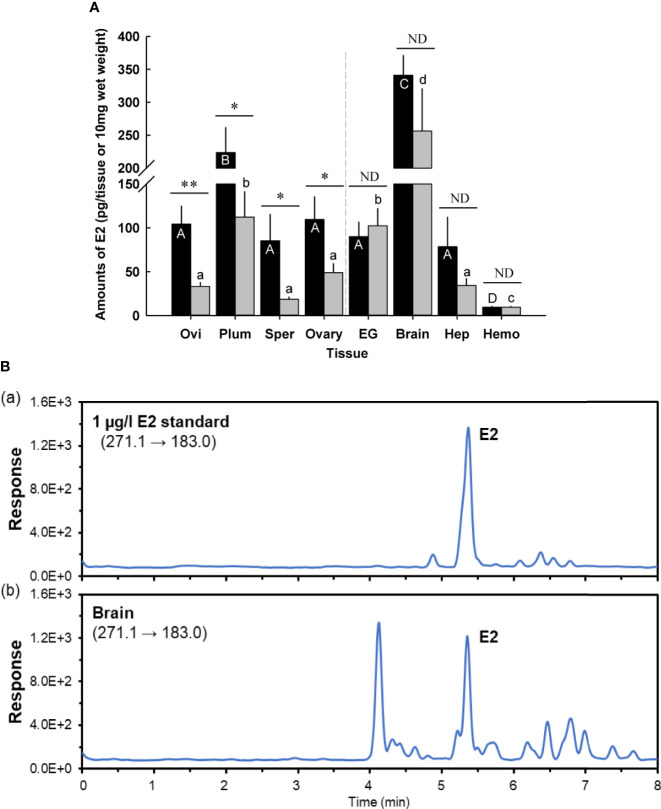
**(A)** Amounts of 17β-estradiol (E2) in prepuberty female *C. sapidus* at early premolt stage (black bar) and late premolt stage (grey bar) based on ELISA analysis. Immature ovaries are examined. Results are presented as the mean ± 1SE pg/EG, brain or 10 mg wet weight (*n* = 5). *p < 0.05; **p < 0.01; nd, no difference. Means within group followed by different letters were different *p* < 0.05. Ovi, ovigerous setae; Plum, plumose setae; Sper, spermathecae; EG eyestalk ganglia; Hep, hepatopancreas and Hemo, hemolymph. **(B)** Representative LC-MS/MS chromatograms of E2 in **(B.a)** a 1 μg/l standard, **(B.b)** extract from the brains (prepuberty female). E2 was confirmed to be present in the brain, but the measured concentration was below the method quantitation limit.

Tissues of the prepuberty females at early and late premolt were measured for E2 using a competitive ELISA (**Figure 1A**). All tissues at both molt stages had E2, ranging from the lowest level in the hemolymph (9.5 ± 1.4 pg/10 μl, *n* = 5) to the highest level in the brain (341.0 ± 30.5 pg/tissue, *n* = 5) at early premolt and from 9.6 ± 1.2 pg/10 μl (hemolymph) to 256.4 ± 64.6 pg/10 mg wet weight (brain) at late premolt (*n* = 5).

E2 levels differed by molt stage in the following tissues: ovigerous and plumose setae, spermathecae, and ovary. Ovigerous setae at late premolt had E2 levels of 33.1 ± 5.0 pg/10 mg wet weight (*n* = 5), significantly lower (*p* < 0.01) than early premolt (104.3 ± 20.9 pg/10 mg wet weight, *n* = 5). The E2 content in plumose setae and ovary at late premolt significantly decreased (*p* < 0.05) by about twofold to 112.3 ± 2.0 pg/10 mg wet weight (*n* = 5) and 49.1 ± 2.2 pg/10 mg wet weight (*n* = 5), respectively, compared to those at early premolt. Significantly (*p* < 0.05) reduced levels of E2 were also observed in the spermathecae (early premolt: 85.4 ± 30.4 pg/10 mg wet weight; late premolt: 18.7 ± 4.6 pg/10 mg wet weight, *n* = 5). However, no changes were identified for the E2 content of the eyestalk ganglia, brain, hepatopancreas, and hemolymph at early and late premolt.

### LC-MS/MS spectrometry

According to the ELISA results, the brain contained the highest E2 level. Therefore, this tissue was chosen for further confirmation of E2 *via* LC-MS/MS. The LC-MS/MS chromatograms for an E2 standard and the brain samples are shown in **Figure 1B**. E2 was positively detected in the brain tissues; however, the measured amount was below the method quantitation limit (*i.e.*, 150 pg/tissue, n =3).

### Sequence analyses of steroidogenesis genes

**Figure 2:**
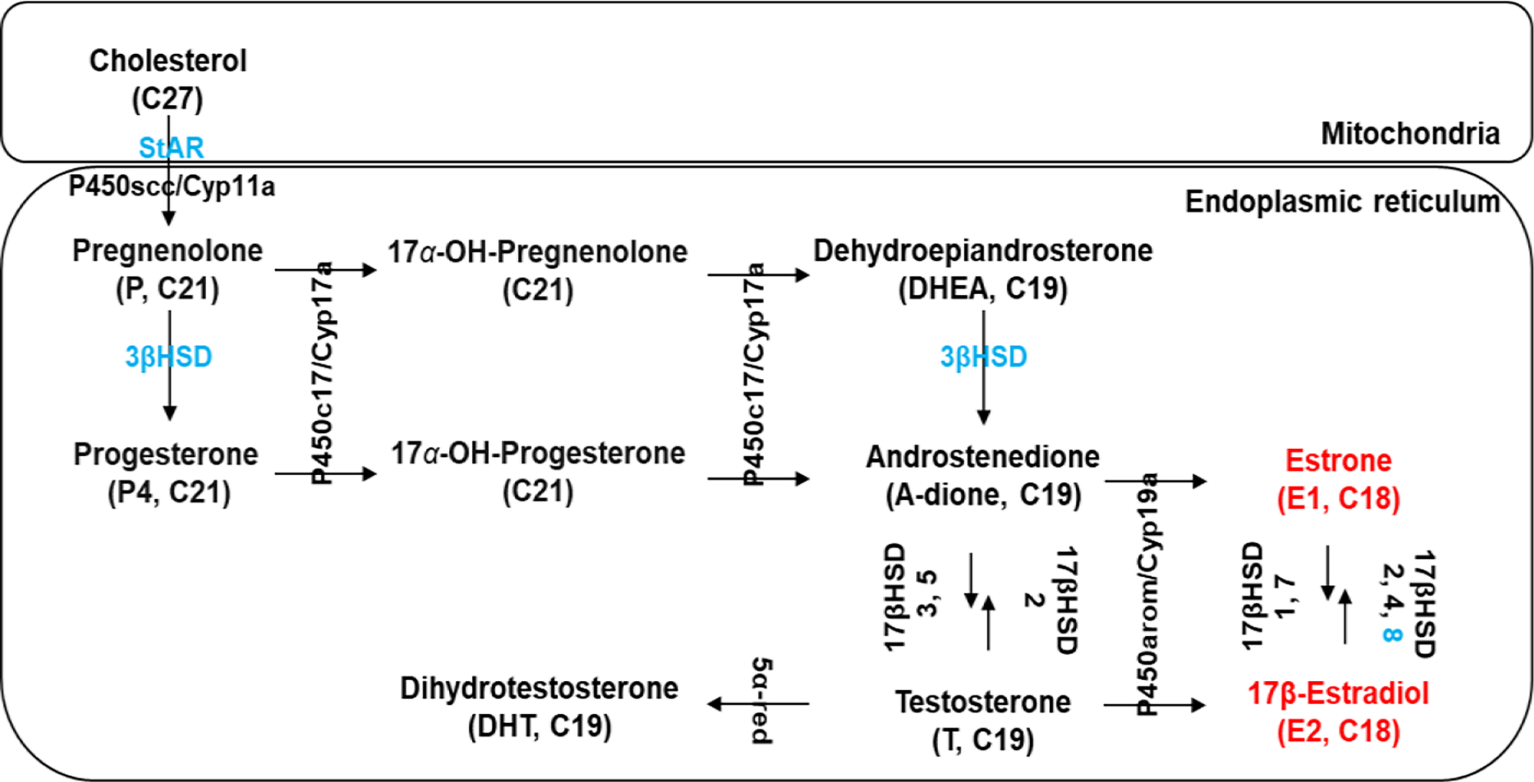
Schematic diagram modified from Janer and Porte (14) showing the presence of several putative steroidogenic genes (marked in blue) in *C. sapidus* known to be involved in vertebrate steroidogenesis. Estrogens, 17β-estradiol (E2) and estrone (E1), are marked in red. StAR, StAR-related lipid transfer protein; P450scc/Cyp11a, P450 side-chain cleavage; 3βHSD, 3β-hydroxysteroid dehydrogenase; 17βHSD, 17β-hydroxysteroid dehydrogenase; P450c17/Cyp17a1, 17α-hydroxylase/17, 20-lyase; P450arom/Cyp19a, P450 aromatase; 5α-red, 5α-reductase.

**Figure 3:**
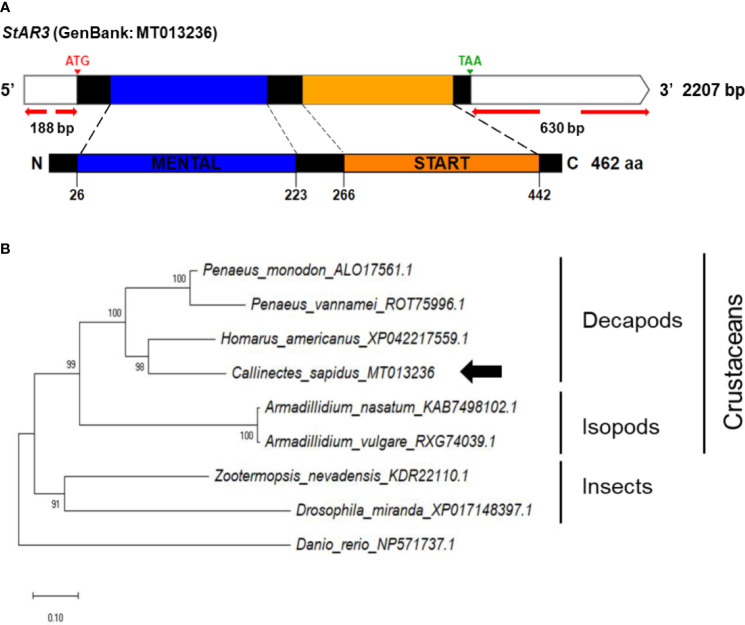
**(A)** Schematic representation of *C. sapidus StAR3.* The conserved domains of StAR3 protein are shown in different colors (MENTAL: cholesterol-capturing domain; START: StAR-related transfer domain). **(B)** Phylogenetic tree of StAR3 was constructed using neighbor-joining (NJ) approach in MEGAX. The amino acid sequences of StAR3 of crustaceans, insects and zebrafish were retrieved from NCBI database. Bootstrap consisted of 1000 replicates.

**Figure 2** shows the potential functions of the steroidogenesis genes, *StAR3*, *3βHSD*, 17*βHSD8a*, 17*βHSD8b*, and *ERR*, in *C. sapidus* involved in the biosynthetic pathway of vertebrate steroid hormones. The full-length *StAR3* and *3βHSD* sequences were cloned from the eyestalk using PCR with gene-specific primers (**Table 1**). *StAR3* cDNA was 2207-bp in length with a 188-bp 5’UTR, a 1389-nt open reading frame (ORF), and a 630-bp 3’UTR with a poly-A tail (**Figure 3A**). The ORF encoded a polypeptide of 462-aa, including a 198-aa cholesterol-capturing domain (MENTAL) and a 177-aa StAR-related transfer domain (START). Multiple alignment analysis showed 32-68% (MENTAL) and 32-38% (START) homologous between vertebrates and invertebrates (**Figure S1A**).

**Figure 4:**
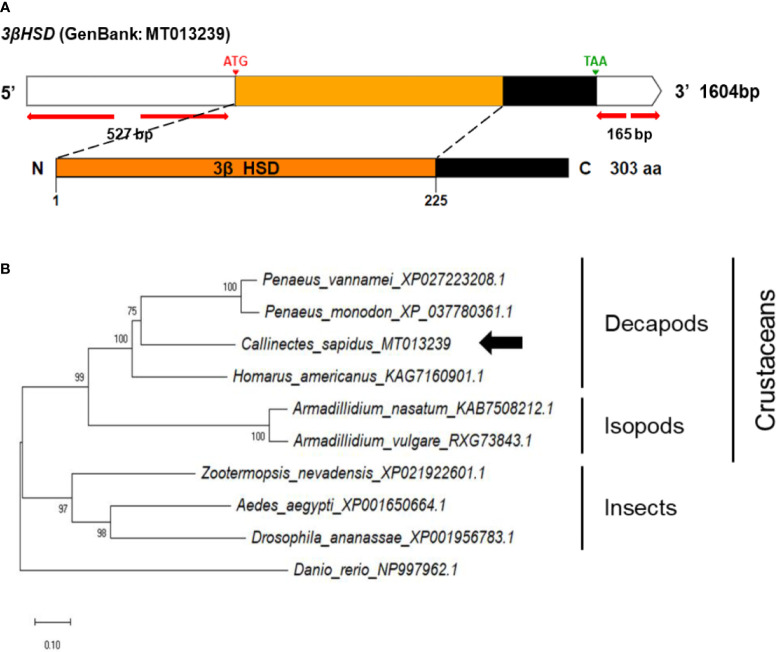
**(A)** Schematic representation of *C. sapidus 3βHSD*. The conserved domain of 3βHSD protein is marked in orange (3β_HSD: 3-beta hydroxysteroid dehydrogenase/isomerase). **(B)** Phylogenetic tree of 3βHSD was constructed using neighbor-joining (NJ) approach in MEGAX. The amino acid sequences of 3βHSD of crustaceans, insects and zebrafish were retrieved from NCBI database. Bootstrap consisted of 1000 replicates.

The full-length *3βHSD* cDNA (1604 nt) consisted of a 527 bp 5’UTR, a 912 bp ORF, and a 165 bp 3’UTR, including the poly-A tail (**Figure 4A**). The conserved domain database identified the deduced amino acid regions from M1 to P225 as the *3βHSD*/isomerase family (3β_HSD), which illustrated 28-60% homology to vertebrates and invertebrates (**Figure S1B**).

**Figure 5:**
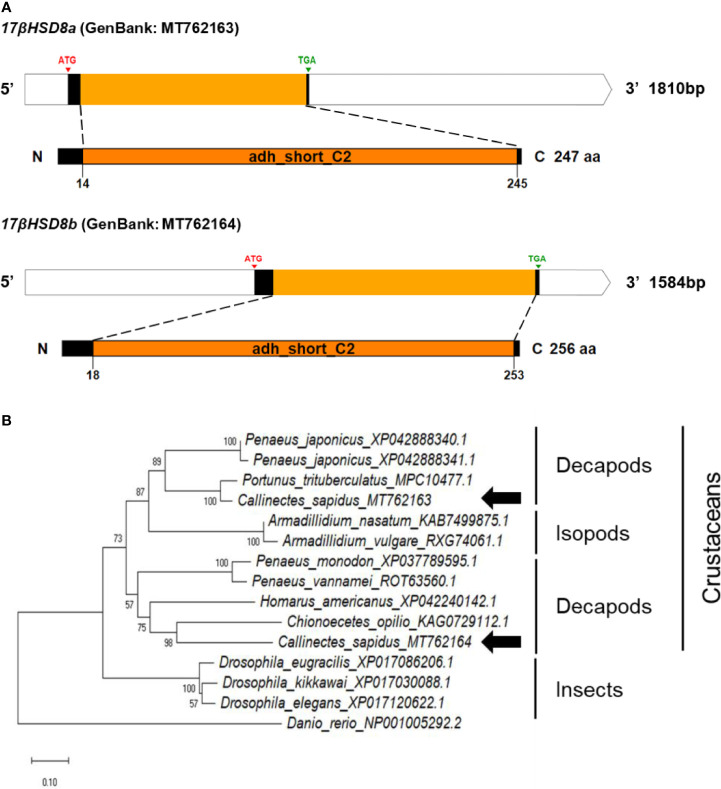
**(A)** Schematic representation of *C. sapidus 17βHSD8*. The conserved domains of 17βHSD proteins are marked in orange (adh_short_C2: Enoyl-(Acyl carrier protein) reductase). **(B)** Phylogenetic tree of 17βHSD8 proteins was constructed using neighbor-joining (NJ) approach in MEGAX. The amino acid sequences of 17βHSD8 of crustaceans, insects and zebrafish were retrieved from NCBI database. Bootstrap consisted of 1000 replicates.

The cDNA sequences of the *17βHSD8a* and *17βHSD8b* genes were isolated, encoding 247 and 256 aa, respectively (**Figure 5A**). Comparison of the conserved domain sequences of *17βHSD8a* and *17βHSD8b* derived by comparing sequences with vertebrates and invertebrates showed 49-91% and 45-50% similarity, respectively (**Figure S1C**). Furthermore, 51% sequence identity was found when the amino acid sequence of *17βHSD8a* ORF was compared with that of *17βHSD8b*.

**Figure 6:**
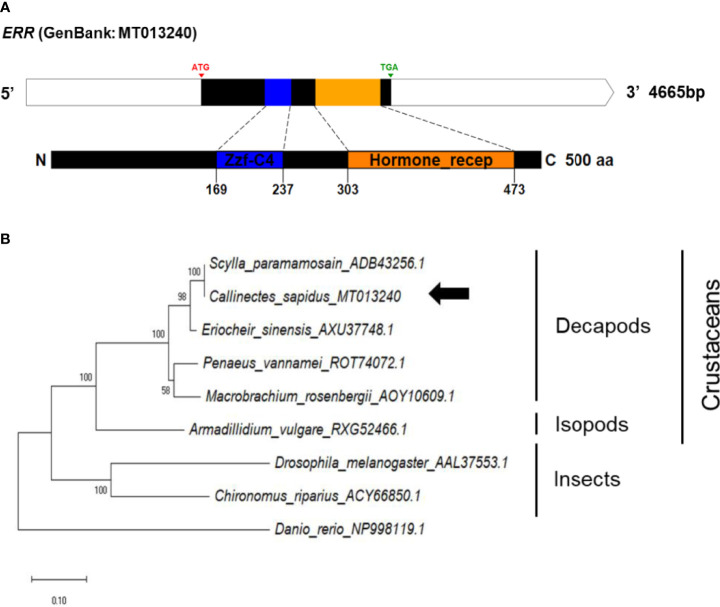
**(A)** Schematic representation of *C. sapidus ERR*. The conserved domains of ERR protein are shown in different colors (zf-C4: zinc finger, C4 type; hormone receptor: ligand-binding domain of nuclear hormone). **(B)** Phylogenetic tree of ERR was constructed using neighbor-joining (NJ) approach in MEGAX. The amino acid sequences of ERR of crustaceans, insects and zebrafish were retrieved from NCBI database. Bootstrap consisted of 1000 replicates.

The *ERR* cDNA contained a 1482-bp ORF encoding 493 aa, consisting of two conserved domains, namely zf-C4 (zinc finger, C4 type) and hormone receptor (ligand-binding domain of nuclear hormone) (**Figure 6A**). The amino acid sequences of conserved regions exhibited 79-90% and 36-90% sequence identities to those of vertebrates and invertebrates, respectively (**Figure S1D**).

### Phylogenetic analysis

The phylogenetic trees of *StAR3*, *3βHSD*, *17βHSD8*, and *ERR* showed that all of the amino acid sequences of *C. sapidus* were clustered with the invertebrate (crustaceans and insects) clade and separated from the vertebrate (zebrafish) clade (**Figures 3**–**6B**). The *C. sapidus StAR3* most closely aligned with *Homarus americanus* and then formed the decapod subclade (100% support) with two shrimp species (**Figure 3B**). The *C. sapidus 3βHSD* was first clustered with *P. monodon* and *P. vannamei*, grouped with *H. americanus*, and then merged into a large branch with isopods, clearly distinguishable from insects and a vertebrate (**Figure 4B**). All of the crustacean *17βHSD8* proteins formed a subclade, in which the *C. sapidus 17βHSD8a* and *17βHSD8b* were positioned close to the *17βHSD8* of two crab species, *P. trituberculatus* and *Chionoecetes opilio*, respectively (**Figure 5B**). The *ERR* protein from *C. sapidus* had the closest evolutionary relationship with *S. paramamosain* and was classified into an invertebrate clade with those isopods and insects (**Figure 6B**).

### Tissue distribution of steroidogenesis genes

**Figure 7:**
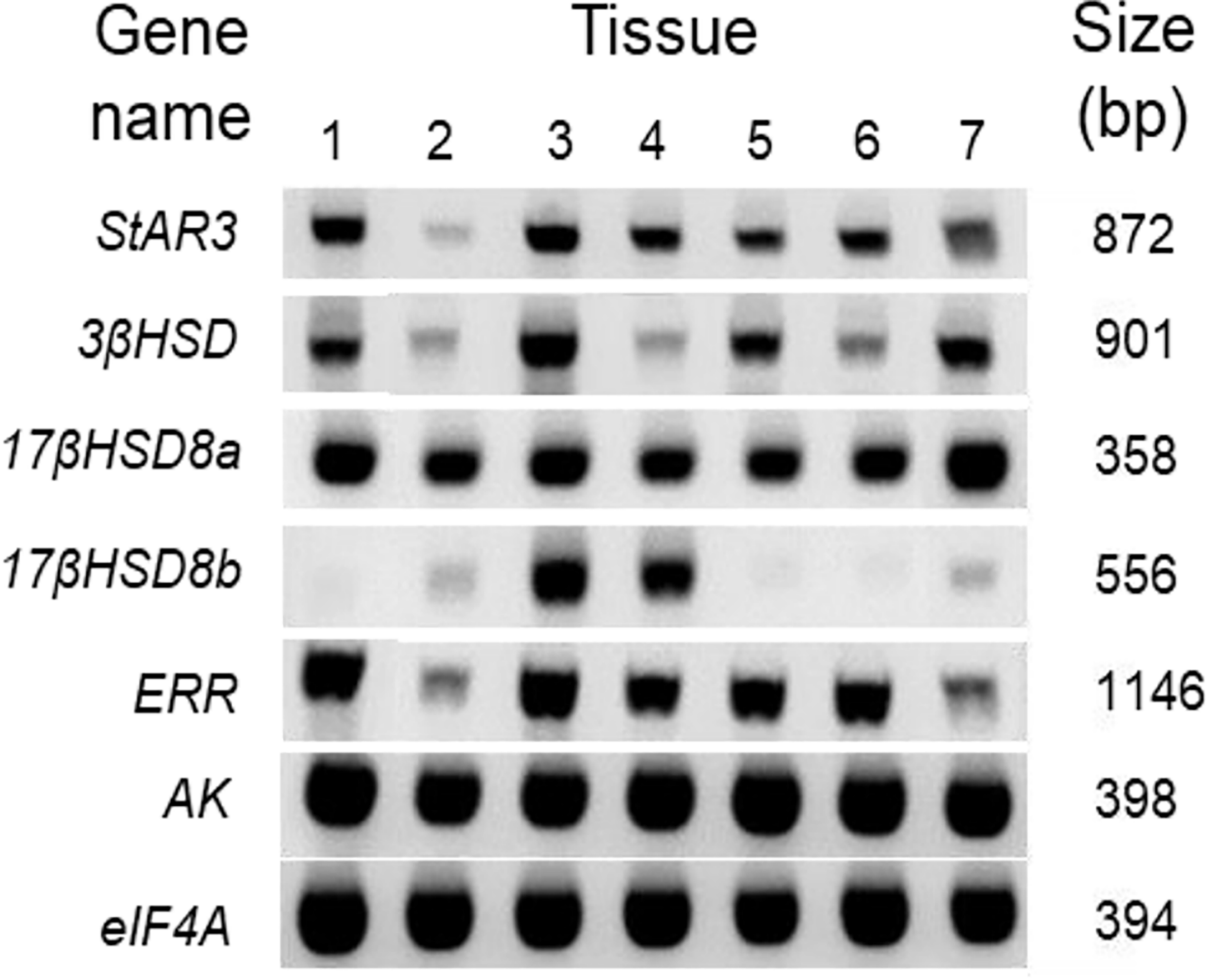
Spatial distribution of vertebrate steroidogenic genes in the tissues of an adult female *C. sapidus*. Each cDNA tissue sample containing 12.5 ng total RNA equivalent was amplified by PCR. The PCR products were analyzed on 1.5% agarose gel and stained with ethidium bromide. The tissues are noted as: 1 = eyestalk ganglia, 2 = hepatopancreas, 3 = brain, 4 = ovary, 5 = spermathecae, 6 = ovigerous setae, 7 = plumose setae.

The spatial distributions of steroidogenesis genes were examined in the various tissue cDNAs of an adult female *C. sapidus* using an endpoint RT-PCR assay (**Figure 7**). All of the tissues showed the presence of *StAR3*, *3βHSD*, *17βHSD8a*, and *ERR*, and the expression of these genes was relatively high in the eyestalk ganglia, brain, and the adult-female-specific tissues but low in the hepatopancreas. In contrast, *17βHSD8b* was mainly expressed in the brain and ovary. *AK* and *eIF4A* had high expression in all tissues.

### Localization of 17βHSD8 transcripts and protein in the ovary

**Figure 8:**
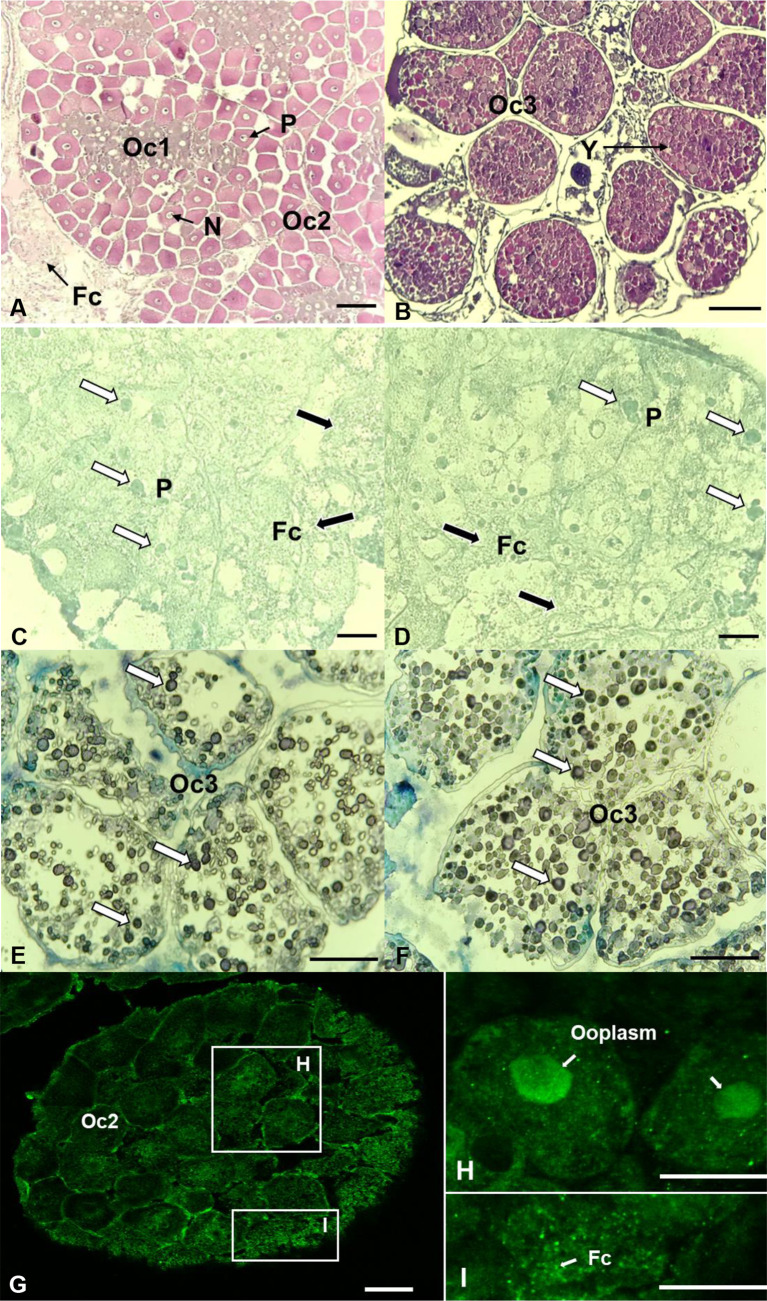
Localization of 17βHSD8 in the ovaries (stage 2 and 3) of adult female *C. sapidus* using *in situ* hybridization (mRNA) and immunohistochemistry (protein). H&E staining **(A, B)**; ISH: antisense probe hybridization (**C–E**: *17βHSD8a*; **D–F**: *17βHSD8b*); Whole-mount immunohistochemistry: 17βHSD8 specific antibody **(G–I)**. The arrows point to a representative positive signal. Abbreviations: N, nucleus; Y, yolk; P, perinucleus; Fc, follicular cell; Oc1, early primary oocyte; Oc2, late primary oocyte; Oc3, early vitellogenic oocyte. Scale bars = 100 μm.

The ovary contained relatively high mRNA levels of *17bHSD8*. Hence, the ovary was used for *in situ* hybridization and immunohistochemistry if the transcripts of *17βHSD8a* and *17βHSD8b* and *17βHSD8* protein were present (ovarian stage 2) (**Figure 8**). The signals of *17βHSD8a* and *17βHSD8b* transcripts were localized in the follicle cells, the periphery of the nuclear membrane of primary oocytes (**Figures 8C, D**), and yolk granules of vitellogenic oocytes (**Figures 8E, F**); furthermore, *17βHSD8* protein was visualized in the follicle cells and ooplasm of primary oocytes (**Figures 8G–I**). Sense probes and pre-immune serum had no positive signals in the ovary (**Figure S2**).

### Effects of CFSH-dsRNA injection on E2 and steroidogenesis gene transcript levels

#### E2

**Figure 9:**
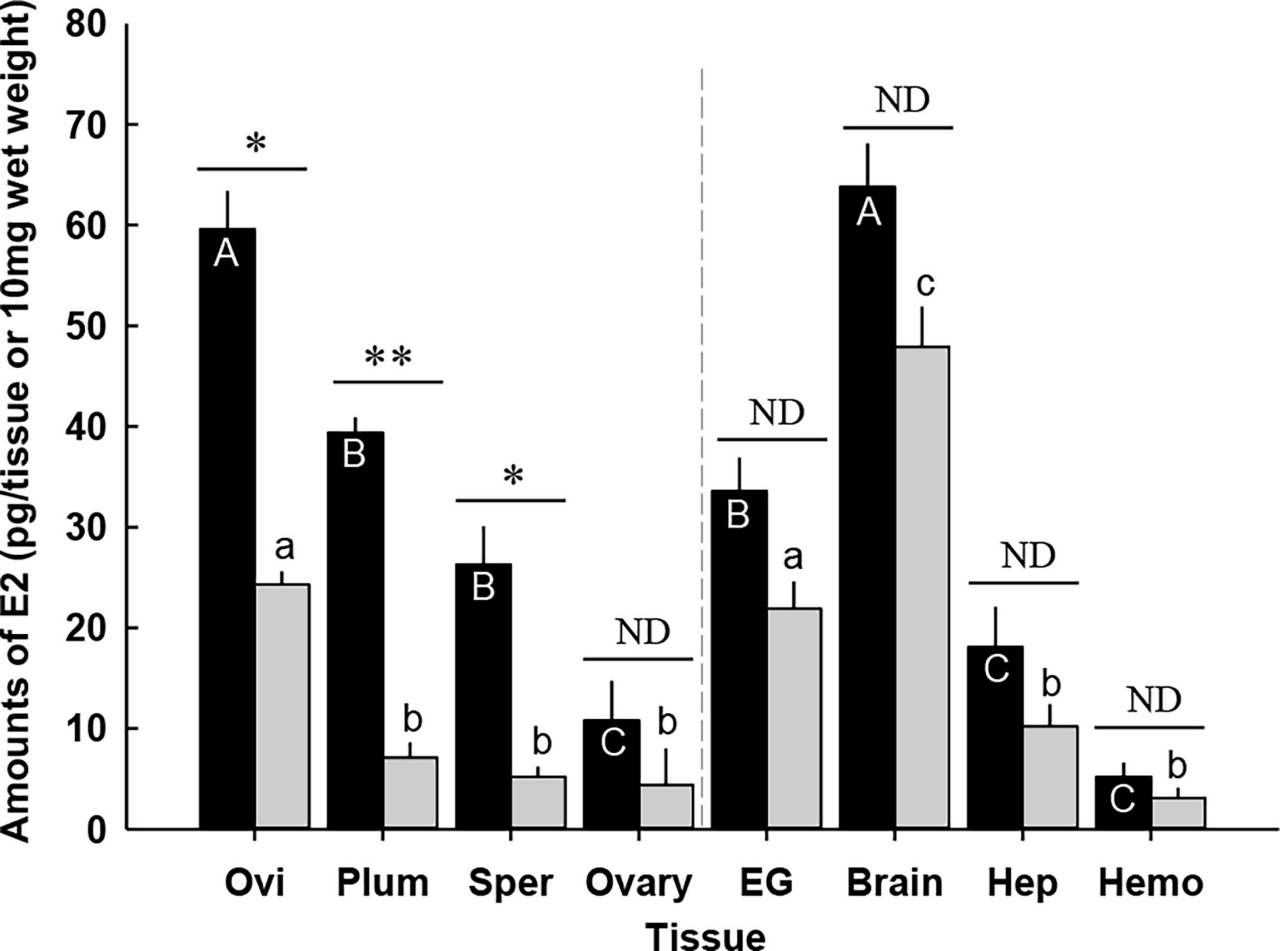
Amounts of 17β-estradiol (E2) in the tissues of the adult female *C. sapidus* at ovarian stage 2 (black bar: saline-injected group; grey bar: CFSH-dsRNA-injected group). Results are presented as the mean ± 1SE pg/EG, brain or 10 mg wet weight (*n* = 5). The data are analyzed two ways for statistical significance: Student’s t-test was calculated when two groups were compared. A non-parametric Kruskal-Wallis test followed by Dunn’s *post-hoc* test was used for multiple group comparisons (letter or *p < 0.05; **p < 0.01; nd, no difference). Ovi, ovigerous setae; Plum, plumose setae; Sper, spermathecae; EG eyestalk ganglia; Hep, hepatopancreas and Hemo, hemolymph.

In general, the effect of CFSH-dsRNA injection on E2 levels differed by tissue (**Figure 9**). E2 content was most significantly reduced in the plumose setae of CFSH-dsRNA injected animals with 7.1 ± 1.5 pg/10 mg wet weight (*n* = 5) compared to controls (39.4 ± 1.5 pg/10 mg wet weight, *n* = 5). The ovigerous setae of CFSH-dsRNA injected females had significantly lower E2 of 24.3 ± 1.3 pg/10 mg wet weight (*n* = 5) than controls, 59.6 ± 3.8 pg/10 mg wet weight (*n* = 5). Moreover, CFSH-dsRNA injection significantly (*p* < 0.05) decreased E2 concentrations in the spermathecae (5.2 ± 1.0 pg/10 mg wet weight, *n* = 5) compared to controls (26.3 ± 3.8 pg/10 mg wet weight, *n* = 5).

CFSH-dsRNA injection slightly reduced E2 levels in the ovary, eyestalk ganglia, brain, hepatopancreas, and hemolymph, but the differences were not statistically different. The brains of the females that received CFSH-dsRNA had E2 levels of 48.5 ± 4.2 pg/tissue (*n* = 5), whereas controls contained 63.8 ± 4.3 pg/tissue (*n* = 5). E2 levels in the hemolymph were as follows: CFSH-dsRNA injection, 3.8 ± 1.2 pg/10 μl; controls, 5.4 ± 1.4 pg/10 μl, *n* = 5).

#### Steroidogenesis gene transcripts

**Figure 10:**
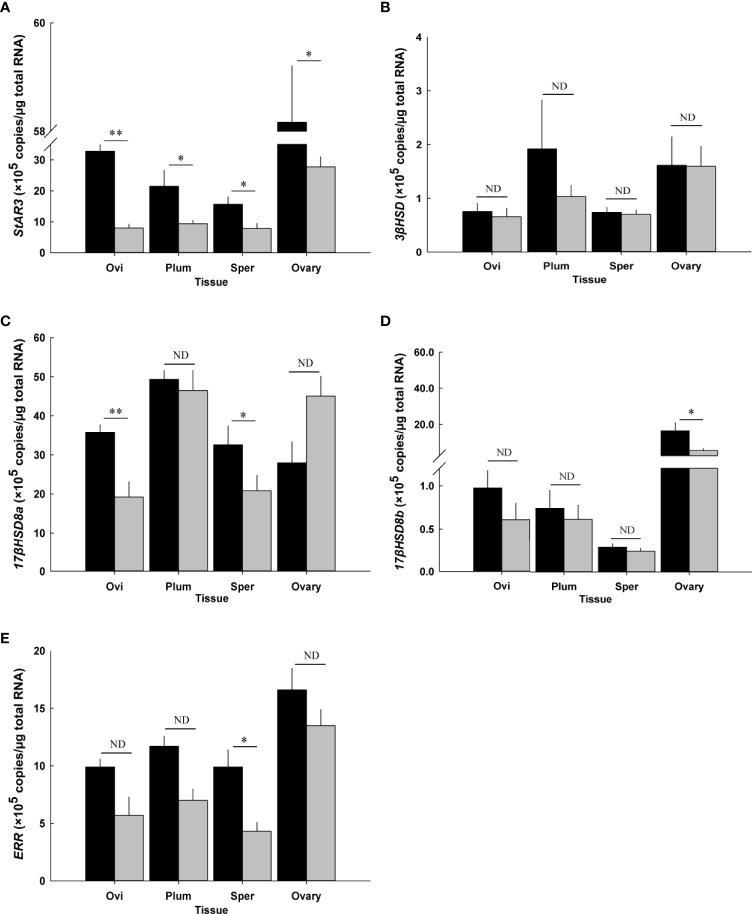
Effect of CFSH-dsRNA injection on the expression of steroidogenesis-related genes using qRT-PCR assay. **(A)** StAR3, **(B)** 3βHSD, **(C)** 17βHSD8a, **(D)** 17βHSD8b, and **(E)** ERR. Expression levels are represented as mean ± SE (*n* = 5) transcripts/μg total RNA. The data are presented as mean ± SE (*n* = 5). *p < 0.05; **p < 0.01; nd, no difference. Ovi, ovigerous setae; Plum, plumose setae; Sper, spermathecae.

The effect of CFSH-dsRNA injection on the transcript levels of steroidogenesis genes was determined using a RT-qPCR assay (**Figure 10**). *CFSH-dsRNA* injection reduced *CFSH* transcripts by 99% to 2.8 ± 0.2 × 10^4^ transcripts/μg total RNA (*n* = 5) compared to the controls with 2.6 ± 0.8 × 10^6^ transcripts/μg total RNA (*n* = 5). The animals injected with CFSH-dsRNA had significantly (*p* < 0.05) reduced *StAR3* transcripts in all examined adult-female-specific tissues compared to controls (**Figure 10A**). The greatest reduction of *StAR3* was observed in the ovigerous setae with 8.0 ± 1.3 × 10^5^ transcripts/μg total RNA (*n* = 5) compared to controls (32.8 ± 2.1 × 10^5^ transcripts/μg total RNA, *n* = 5).

The ovigerous setae and spermathecae from CFSH-dsRNA injected animals had significantly (*p* < 0.05) lower *17βHSD8a* by about 50%, namely 19.2 ± 3.9 × 10^5^ transcripts/μg total RNA (*n* = 5) and 20.8 ± 4.0 × 10^5^ transcripts/μg total RNA (*n* = 5) compared to controls with 35.7 ± 2.0 × 10^5^ transcripts/μg total RNA (*n* = 5) and 32.6 ± 4.9 × 10^5^ transcripts/μg total RNA (*n* = 5), respectively (**Figure 10C**). After CFSH-dsRNA injection, statistically significant (*p* < 0.05) downregulation of *17βHSD8b* and *ERR* expression was only observed in the ovary (CFSH-dsRNA injection: 5.3 ± 1.1 × 10^5^ transcripts/μg total RNA; controls: 16.3 ± 4.6 × 10^5^ transcripts/μg total RNA, *n* = 5, **Figure 10D**) and spermathecae (CFSH-dsRNA injection: 4.3 ± 0.8 × 10^5^ transcripts/μg total RNA; controls: 9.9 ± 1.5 × 10^5^ transcripts/μg total RNA, *n* = 5, **Figure 10E**), respectively. No significant (*p* > 0.05) changes in *3βHSD* were observed (**Figure 10B**).

## Discussion

The present study described the presence and concentration of E2 in tissues of prepuberty and adult females of blue crab *C. sapidus*, the isolation of cDNA sequences and transcript levels of several vertebrate steroidogenesis genes related to E2, the effects of *CFSH* on E2, and expression analysis of steroidogenesis genes.

### The presence of E2 in prepuberty and adult females

The presence of E2 in the crustacean tissues has been primarily reported using antibody-based assays. Our ELISA results showed that various tissues of the prepuberty and adult blue crab females contain E2, congruent with earlier findings that report E2 in the hemolymph, hepatopancreas, and ovary of *H. americanus* (31), *Pandalus kessleri* (32), and *Artemia* sp. (33). In this study, an LC-MS/MS spectrometry method confirmed that E2 measured in blue crab tissues is the genuine and endogenous E2. The optimized LC-MS/MS analytical protocol exhibited a good recovery (89%), and the measured E2 concentration matched that from the ELISA analysis. Due to the higher method quantitation limit of the LC-MS/MS method for E2, ELISA was more preferred for E2 measurement in *C. sapidus*.

This study focused on E2 in early and late premolt *C. sapidus* females at prepuberty, during which time the ovigerous and plumose setae are associated with development of blue crab females (19). These adult-specific tissues are developing at early premolt and are fully developed at late premolt. Our data showed a marked difference in E2 levels in a molt stage-specific manner, with higher levels at early premolt than at late premolt, when these tissues primarily develop the features specific to adults for mating and maternal care systems. Based on previous findings that CFSH is required to develop adult-specific morphological features, it is reasonable to suggest that higher E2 levels in these tissues may be linked to CFSH function.

In both prepuberty and adult females, maximum E2 levels were present in the brain. In vertebrates, E2 affects the structure and function of the nervous system (34) and provides positive feedback on the development and function of the gonadotropic axis (35–37). The function of E2 in the brain of crustaceans is poorly understood. Considering that mating occurs immediately at the onset of puberty-terminal molting, E2 in the brain of the prepuberty females may be linked with sexual behaviors and involved in the reproductive neuroendocrine responses that occur in the brain of crustaceans (38).

In comparison to the vitellogenic ovaries (ovarian stage 2) in adult females, the undifferentiated ovaries in prepuberty females had higher E2 levels. This finding appears to be rather different from those reported in other decapods, in which E2 levels in the ovary significantly increased with ovarian development and oocyte maturation (7, 39, 40). One plausible explanation is that season affects E2 synthesis in female gonads. In the present study, ovaries were sampled from the prepuberty and adult females during summer and winter months, respectively. Thus, warmer temperatures may stimulate reproductive activities and biosynthesis of E2, while these processes are slowed or halted in colder temperatures.

The levels of E2 measured in the tissues were converted and presented as pg/tissue type, with the same 10 mg wet weight wherever possible. In the present study, E2 levels were lowest in the hemolymph of the prepuberty and adult females, but hemolymph accounted for ~15% of body weight (*e.g.*, 70 ± 10 g for prepuberty, 160 ± 20 g for adult, *n* = 10); therefore, the hemolymph contained the greatest amount of E2 compared to other tissues, suggesting that E2 may act as a hormone in this species.

The presence of CFSH protein was consistent throughout the female life cycle. Considering the vital role of CFSH in developing adult female-specific tissues, the following question is asked if this hormone has any functions in these tissues, possibly involving E2. Indeed, our data showed that the tissues specific to adult females contained E2. The E2 levels measured in adult tissues were compensated with the increment of their size through the puberty-terminal molting, *i.e.*, a maximal molt increment of 50%, compared to those of the late premolt stage. Interestingly, the ovigerous setae of adults contained E2 levels similar to those of the late premolt prepuberty females, but the other adult tissues had lower E2 contents than at late premolt, which might stem from the lower E2 in the adult hemolymph.

### Identification of steroidogenesis genes in C. sapidus

To further examine the involvement of putative E2 steroidogenesis genes to confirm the presence of endogenous E2 in female *C. sapidus*, several cDNAs encoding these orthologues were isolated and characterized. The blue crab cDNA sequences of steroidogenesis genes were grouped and formed a clade with other crustacean sequences. The sequences of invertebrates, including insects and crustaceans, were separated from a vertebrate sequence, zebrafish *Danio rerio*, as an out group.

*StAR* was chosen due to its involvement in the translocation of cholesterol from the outer to the inner mitochondrial membrane, a process that is regarded as the first and rate-limiting step in the steroidogenic pathway of vertebrates (41). Therefore, *StAR* plays a vital role in the regulation of steroidogenesis. In this study, we identified the presence of *StAR3* but not *StAR*, indicating that a *StAR*-independent steroidogenic pathway may be adopted by *C. sapidus*. A similar phenomenon was reported in *M. rosenbergi* (17).

*3βHSD* and *17βHSD* regulate two different steps in vertebrate steroidogenesis. The former is responsible for both the reduction and oxidation of the 3-keto/hydroxyl and the Δ^5^-Δ^4^-isomerization that generate progesterone and androstenedione, while the latter is involved in the synthesis of active sex steroids that catalyze androstenedione to testosterone, estrone to E2, or vice versa (14). To date, *3βHSD* and types 2, 3, 4, 6, 8, 11, and 14 of *17βHSDs* were identified in *M. rosenbergii* and *P. trituberculatus* (16, 17). In the present study, a *3βHSD* and two isoforms of *17βHSD8* were identified in *C. sapidus*.

Due to the high structure and sequence similarity and close phylogenetic relationship to ERs, the orphan nuclear receptors, ERRs, were identified (42). Unlike ERs, ERRs are not activated by E2 but bind to anthropogenic estrogenic ligands, estrogen, and ERR response elements and, thus, play a physiological role in embryonic and gonadal development (43, 44). In crustaceans, ERRs were detected in *Daphnia magna* (45), *D. pulex* (46), and *Diaphanosoma celebensis* (47), and may be involved in estrogen signaling and metabolism pathways.

The most important cytochrome P450 enzymes, including the P450scc/cyp11a (P450 side chain cleavage), P450c17/cyp17a, and P450arom/cyp19a (P450 aromatase), were not identified in this study. The presence of P450c17 has been documented in echinoderms (48), mollusks (49), and some decapods (17), while P450scc and P450arom have not yet been identified outside of vertebrates (14). However, the conversion of cholesterol to pregnenolone and aromatase-like activity were detected in some invertebrates (50–53), indicating the orthologues of P450scc and P450arom that function in cholesterol catabolism and estrogen synthesis may also exist in invertebrates.

### Tissue distribution of steroidogenesis genes and localization of 17βHSD8 in the ovary

A broad tissue distribution of vertebrate steroidogenesis genes was found in adult female *C. sapidus* using RT-PCR, possibly explaining the widespread presence of E2. The relatively high expression levels of all steroidogenic genes in the brain were consistent with the ELISA results, wherein the brain had the highest E2 levels among tested tissues. Similarly, most of the enzymes involved in steroidogenesis were highly expressed in the central nervous system of mammals (54), scallops (49), and prawn (17). On the other hand, the low E2 contents in the hepatopancreas may be caused by the relatively low transcript levels of *StAR3* and *3βHSD*, which are related to E2 synthesis, and the high level of *17βHSD8a*, which is associated with E2 metabolism. Interestingly, *17βHSD8* presented two isoforms with different expression patterns: *17βHSD8b* was mainly expressed in the brain and ovary, implying its prominent role in E2 metabolism in these tissues; and, *17βHSD8a* was ubiquitously expressed in all tissues. An extensive tissue distribution pattern of *17βHSD8* was also found in humans (55), mice (56), and scallops (57). Similar to *StAR3*, *ERR* was expressed in all of the examined tissues, and the relatively low levels in the hepatopancreas and plumose setae may corroborate *ERR* involvement in the transcriptional regulation of *StAR* (58).

The localizations of *17βHSD8* mRNAs and protein were visualized by *in situ* hybridization and immunohistochemistry to indirectly support the existence of endogenous E2 in *C. sapidus*. The positive signals of *17βHSD8* transcripts and protein were clearly visible in the follicle cells and oocytes of the ovary, consistent with results from a previous study conducted at both the gene and the protein levels (57). However, positive hybridization signals were also detected in granulosa cells of growing follicles and luteal cells in the mouse ovary (56). We speculate that the discrepancy may be attributed to the differences in ovary structures between mammals and crustaceans. The *17βHSD8* transcripts were obviously visible in the yolk granules of vitellogenic oocytes, implying the physiological role of *17βHSD8* in vitellogenesis and ovarian development. In mollusks, changes in the expression of *17βHSD8* appear to be related to the maturity of female gonads (57). The high transcript abundance of *17βHSD8* and protein levels may be required to maintain the optimal E2 concentrations for oocyte development and ovarian maturation in *C. sapidus.*

### Effects of CFSH-dsRNA injection on E2 and steroidogenesis gene transcript levels

After finding E2 in the adult-specific tissues of adult females, along with the presence of CFSH protein in adult eyestalks (19), the function of CFSH in these tissues was examined with E2 and steroidogenesis genes using CFSH-dsRNA injection. The dsRNA-mediated RNA interference has been used to define the function of a specific protein. In many decapod species, the injection of a gene-specific dsRNA significantly decreased the target transcripts and protein levels (19, 59–62). Therefore, if CFSH function involves E2, CFSH-dsRNA was expected to reduce E2 and steroidogenesis gene transcript levels in these adult-specific tissues compared to controls.

This hypothesis was confirmed by our data, because E2 levels in the adult-specific tissues were lower than in controls, while the E2 contents in other tissues, such as ovary, brain, eyestalk ganglia, hepatopancreas, and hemolymph, exhibited no differences. Among the examined steroidogenesis genes in the control animals, *17βHSD8a* and *StAR3* transcripts were the highest, followed by *ERR*, *3βHSD*, and then *17βHSD8b*. The most consistent effect of CFSH-dsRNA injections was observed with *StAR3* transcripts in the adult-specific tissues, where the levels were lower than controls, congruent with the E2 content in these tissues. It is plausible to suggest that CFSH exerts its functions in the adult female-specific tissues *via* E2 synthesis by regulating the expression of *StAR3*. E2 synthesis in crustaceans is the same as described in vertebrates, wherein StAR is required for cholesterol movement. This step is also known to critical for ecdysteroidogenesis in the Y-organ (63).

CFSH-dsRNA injection had no significant effects on transcript levels of *3βHSD* in adult-female-specific tissues but decreased expression levels of *17βHSD8a, 17βHSD8b*, and *ERR* in a tissue-specific manner, indicating these genes were unlikely to be directly regulated by *CFSH*. Previous studies demonstrated that CFSH might not be involved in ovarian development (19, 64). However, the significantly reduced transcript level of *17βHSD8b* in the ovary of adult females injected with CFSH-dsRNA may affect the metabolic efficiency of E2 leading to changes in ovarian differentiation. The relationship between transcript levels and protein contents are unknown (65, 66); therefore, the long-term effects of CFSH-dsRNA injection on translation of steroidogenesis genes in the adult-female-specific tissues need to be further investigated. Moreover, the broad distribution of testosterone in males is expected based on the presence of E2 and steroidogenic genes in female *C. sapidus*.

### Effect of CFSH-dsRNA injection on the maintenance of adult-female-specific tissues

After a long-term CFSH-dsRNA injection, notable abnormalities were not observed in the length and abundance of ovigerous and plumose setae, wet weight and size of spermathecae, and the presence, position, and size of gonopores (**Figure S3**). These results indicated that CFSH and/or E2 may not be required for the maintenance of brooding and mating systems in adult females. In addition, 30 injections of CFSH-dsRNA seemed to be lethal because ~50% mortality (3/8) was obtained, and no mortality was observed in saline-administrated animals. The difference in mortality might have occurred as off-target effects of CFSH-dsRNA.

## Conclusion

This study reported the presence of E2 in *C. sapidus* using ELISA and LC-MS/MS. E2 and the transcripts of *StAR3*, *3βHSD*, *17βHSD8a*, *17βHSD8b*, and *ERR* were distributed in all examined tissues. The transcripts of *17βHSD8* were visible in the follicle cells, the periphery of the nuclear membrane of primary oocytes, and yolk granules of vitellogenic oocytes, while *17βHSD8* protein was mainly visualized in the follicle cells and ooplasm of primary oocytes. Long-term injection of CFSH-dsRNA significantly reduced E2 levels and specifically *StAR3* expressions in the adult-female-specific tissues, including ovigerous and plumose setae and spermathecae. The results presented here suggested that E2 might be involved in the signal transduction of CFSH in the adult-female-specific tissues that are required for developing brooding and maternal care.

## Data availability statement

The sequencing data are deposited to GenBank, with the following accession numbers: StAR3 (MT013236); 3βHSD (MT013238); 17βHSDa (MT762163); 17βHSDb (MT762164); 583 and ERR (MT013240)).

## Author contributions

TW: Data acquisition, Analysis, Visualization, Writing – Original draft preparation, Writing & Editing. KH: LC-MS/MS analysis and Writing–Review & Editing. LB: LC-MS/MS analysis and Writing–Review & Editing. JSC: Conceptualization, Data acquisition, Project Administration, Resources, Supervision, Validation, Original draft Writing – Review & Editing. All authors contributed to the article and approved the submitted version.

## Funding

This research was supported by an NSF grant (NO. 1146774) to JC and the China Scholarship Council (CSC201906330108) to TW.

## Acknowledgments

We are grateful to the staff of the aquaculture research center (ARC, Institute of Marine and Environmental Technology, Baltimore, MD) for maintaining the closed recirculating system and Zooquatic Laboratory for daily water analysis. All experimental animals were produced in the Chung laboratory (ARC, IMET). The broodstock females for juvenile production were collected onboard Miss Paula in the mid-upper Chesapeake Bay with the help of Captain CJ Candy and his crews. This research was supported by an NSF grant (NO. 1146774) to JC and the China Scholarship Council (CSC201906330108) to TW.

## Conflict of interest

The authors declare that the research was conducted in the absence of any commercial or financial relationships that could be construed as a potential conflict of interest.

## Publisher’s note

All claims expressed in this article are solely those of the authors and do not necessarily represent those of their affiliated organizations, or those of the publisher, the editors and the reviewers. Any product that may be evaluated in this article, or claim that may be made by its manufacturer, is not guaranteed or endorsed by the publisher.

## Supplementary material

Supplementary Figure 1 Multiple sequence alignment of the StAR3 amino acid sequence among species. The conserved functional structures (MENTAL and START domains) are marked in box **(A)**. Multiple sequence alignment of the 3βHSD amino acid sequence among species. The conserved functional structure (3β_HSD domain) is marked in box **(B)**. Multiple sequence alignment of the 17βHSD8 amino acid sequences among species. The conserved functional structure (adh_short_C2 domain) is marked in box **(C)**. Multiple sequence alignment of the ERR amino acid sequence among species. The conserved functional structures (zf-C4 and Hormone_recep domains) are marked in box **(D)**. Identical residues are shown as white letters with red background, and similar residues are shown as black letters with yellow background.

Supplementary Figure 2
*17βHSD8a* **(A, B)** and *17βHSD8b* **(C, D)** sense probe hybridization were used as control of the antisense probe hybridization. Whole-mount immunohistochemistry: pre-immune serum **(E)** was applied as control of the 17βHSD8 specific antibody. Scale bars = 100 μm.

Supplementary Figure 3 No notable abnormalities were observed in the length and abundance of ovigerous **(A*)** and plumose setae **(B*)**, wet weight and size of spermathecae **(C*)**, and the presence, position and size of gonopores **(D*)** after multiple injections of CFSH-dsRNA. Scale bar = 5 mm.
