# Supplementary figures and images for: 17β-Estradiol (E2) may be involved in the mode of crustacean female sex hormone (CFSH) action in the blue crab, Callinectes sapidus
Source: Front Endocrinol (Lausanne). 2022 Jul 25;13:962576. doi: 10.3389/fendo.2022.962576 (PMC9358259; doi:10.3389/fendo.2022.962576)

Wang et al., Figs.S1

| Fig. S1A (StAR3) |
| --- |
| 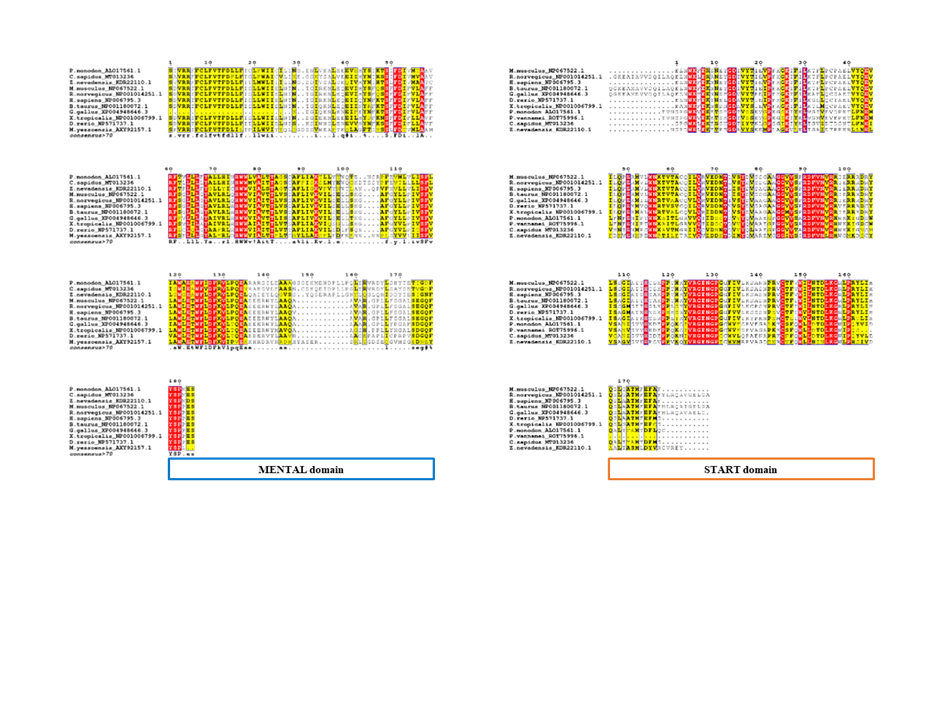 |

| Fig. S1B (3βHSD) |
| --- |
| 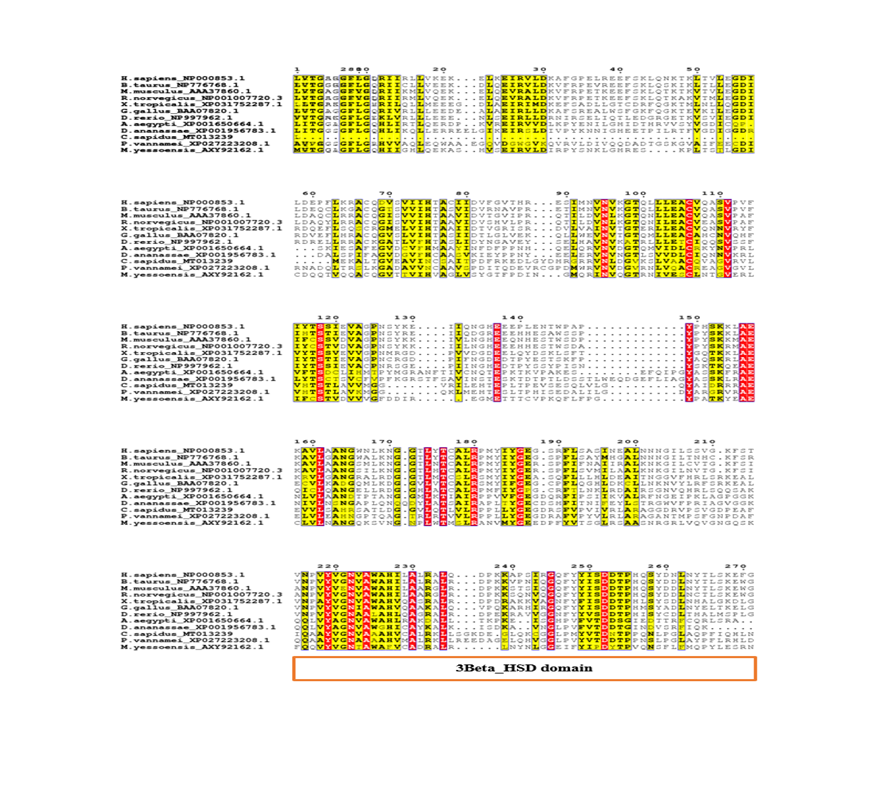 |

| Fig. S1C (17βHSD8) |
| --- |
| 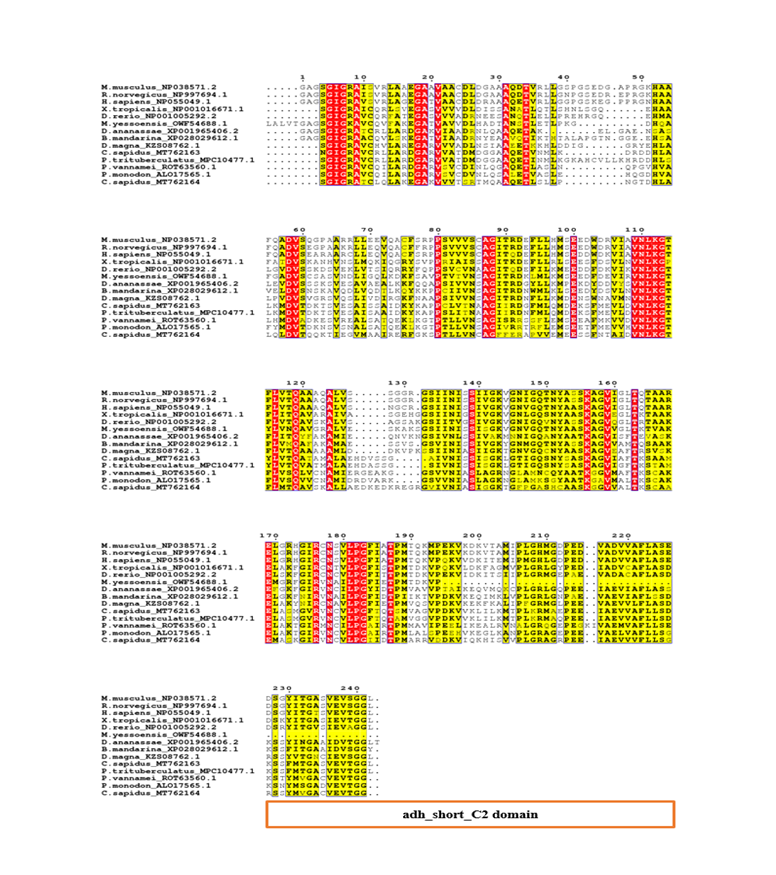 |

| Fig. S1D (ERR) |
| --- |
| 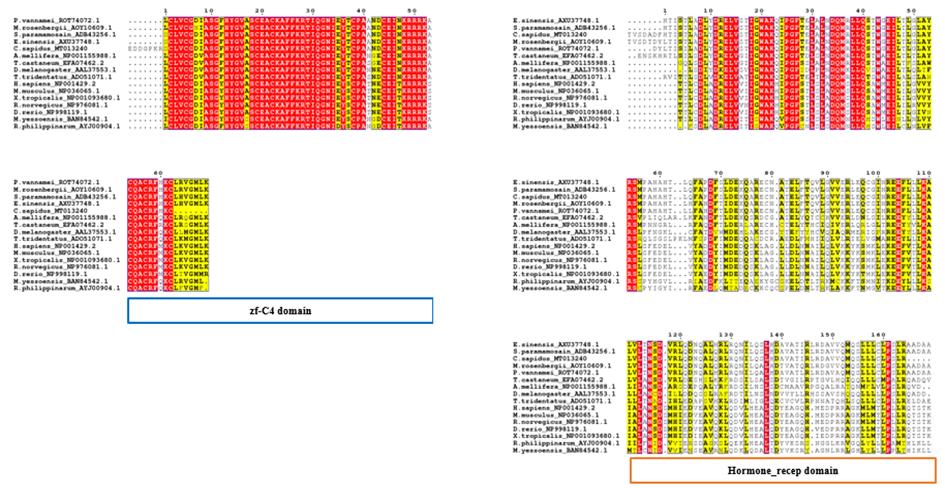 |

Supplement: Supplementary Figure 1 — Multiple sequence alignment of the StAR3 amino acid sequence among species. The conserved functional structures (MENTAL and START domains) are marked in box (A). Multiple sequence alignment of the 3βHSD amino acid sequence among species. The conserved functional structure (3β_HSD domain) is marked in box (B). Multiple sequence alignment of the 17βHSD8 amino acid sequences among species. The conserved functional structure (adh_short_C2 domain) is marked in box (C). Multiple sequence alignment of the ERR amino acid sequence among species. The conserved functional structures (zf-C4 and Hormone_recep domains) are marked in box (D). Identical residues are shown as white letters with red background, and similar residues are shown as black letters with yellow background. [file DataSheet_1.docx]

Fig.S2

| 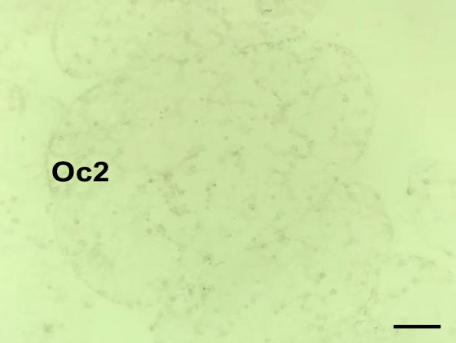  A | 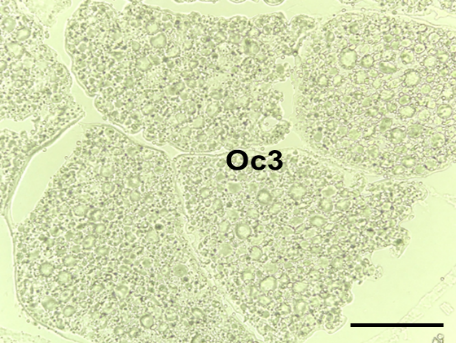  B |  |
| --- | --- | --- |
| 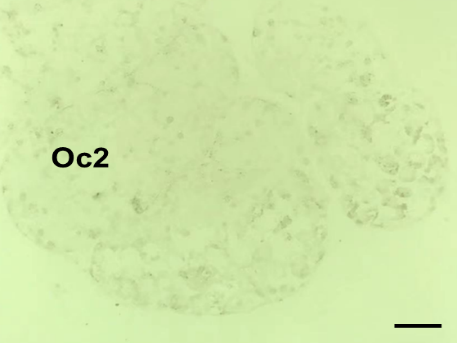  C | 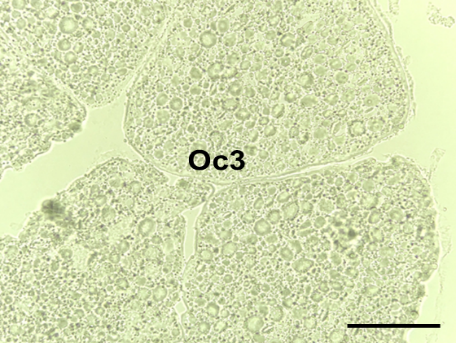  D | 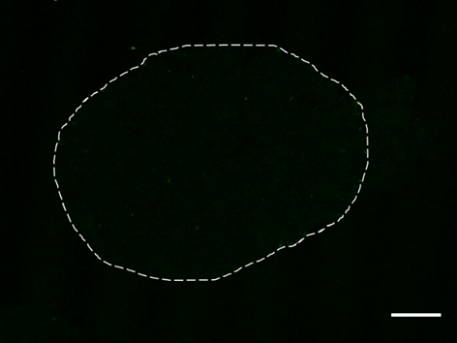  E |

Supplement: Supplementary Figure 2 — 17βHSD8a (A, B) and 17βHSD8b (C, D) sense probe hybridization were used as control of the antisense probe hybridization. Whole-mount immunohistochemistry: pre-immune serum (E) was applied as control of the 17βHSD8 specific antibody. Scale bars = 100 μm. [file DataSheet_2.docx]

Fig. S3

| Saline-injected adult females | *CFSH-dsRNA*-injected adult females |
| --- | --- |
| 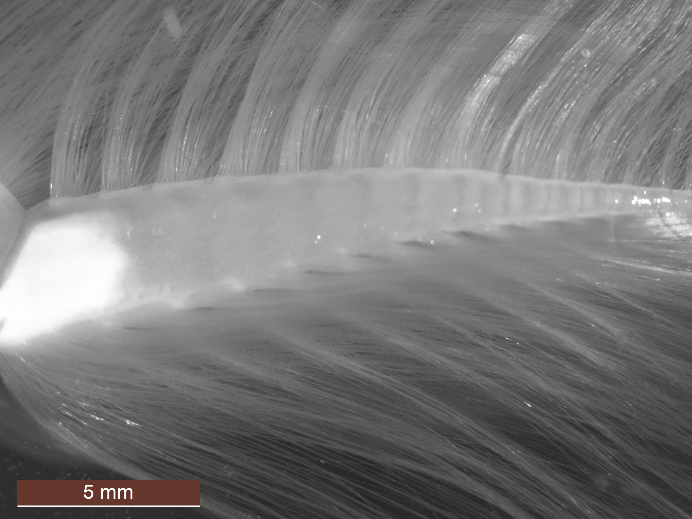  **A** | 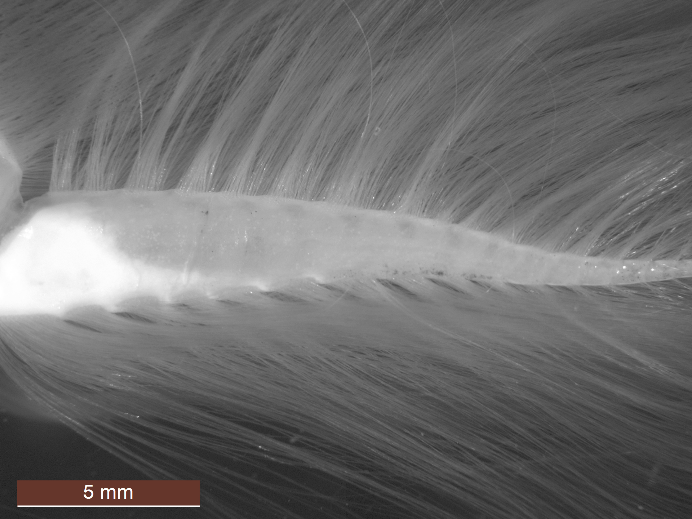  **A*** |
| 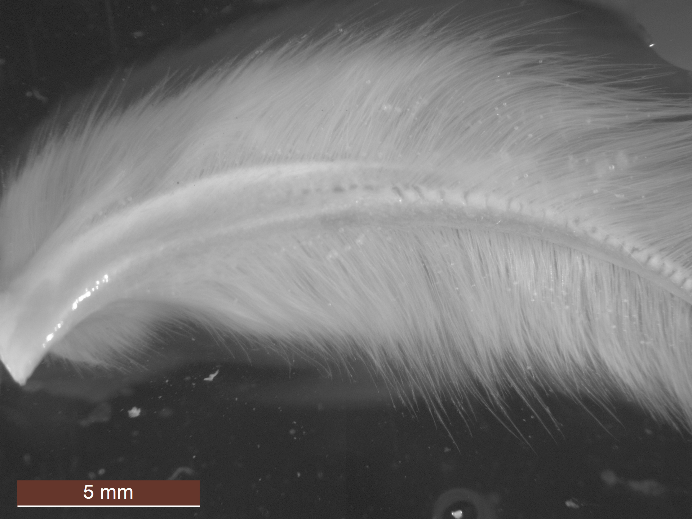  **B** | 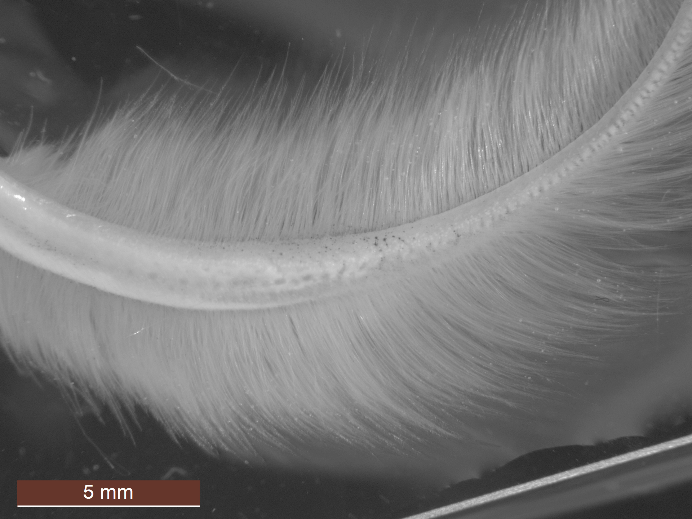  **B*** |
| 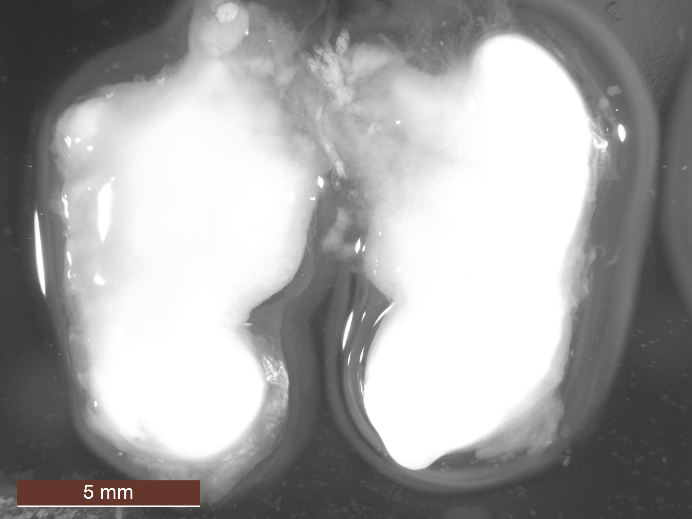  **C** | 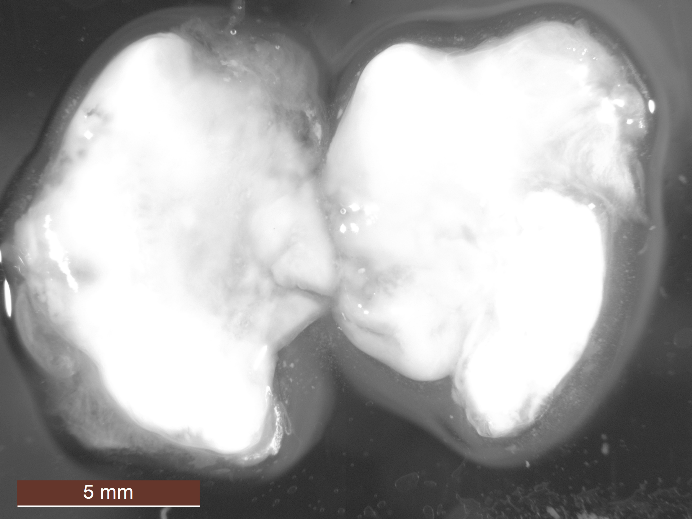  **C*** |
| 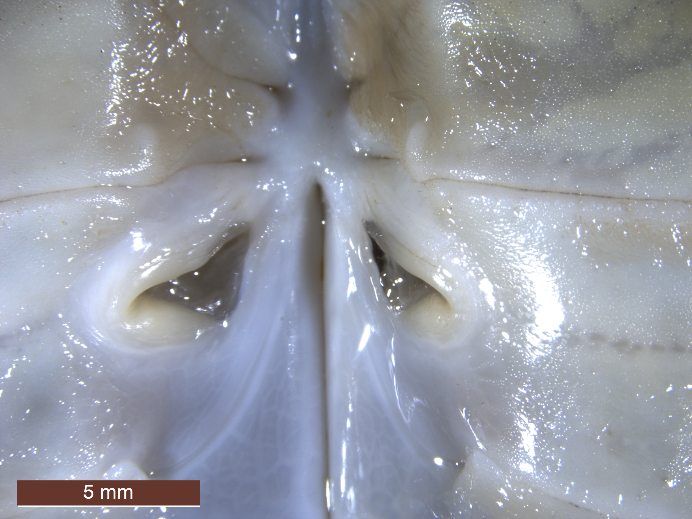  **D** | 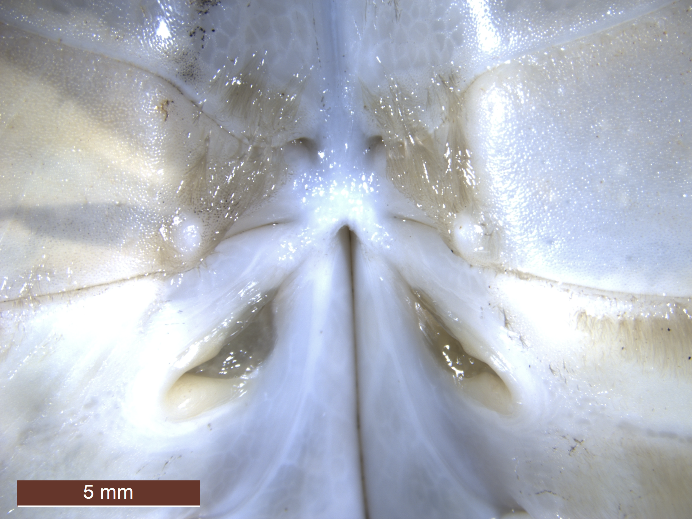  **D*** |
|  |  |

Supplement: Supplementary Figure 3 — No notable abnormalities were observed in the length and abundance of ovigerous (A*) and plumose setae (B*), wet weight and size of spermathecae (C*), and the presence, position and size of gonopores (D*) after multiple injections of CFSH-dsRNA. Scale bar = 5 mm. [file DataSheet_3.docx]
