# Supplementary material for: 17β-Estradiol (E2) may be involved in the mode of crustacean female sex hormone (CFSH) action in the blue crab, Callinectes sapidus
Source: Front Endocrinol (Lausanne). 2022 Jul 25;13:962576. doi: 10.3389/fendo.2022.962576 (PMC9358259; doi:10.3389/fendo.2022.962576)
Supplement: Supplementary file 4 [file Table_1.docx]

Table S1. The MS/MS operational parameters and method performance metrics for E2, E2-d3, and EE2-d4.

| Analyte | Ion transition ^a^ | Collison energy  (V) | Linear range  (µg/l) | R^2^ | MDL^b^  (ng/g) | MQL^b^  (ng/g) | Internal standard |
| --- | --- | --- | --- | --- | --- | --- | --- |
| E2 | 271.1 → 183.0 | -44 | 1–100 | 0.993 | 1.5 | 5.0 | EE2-d_4_ |
|  | *271.1 → 145.1* | -42 |  |  |  |  |  |
| E2-d3 | 274.2 → 185.1 | -45 | 1–100 | 0.991 | 1.5 | 5.0 | EE2-d_4_ |
|  | *274.2 → 145.1* | -43 |  |  |  |  |  |
| EE2-d4 | 299.1 → 147.0 | -46 | - | - | - | - | - |
|  | *299.1 → 161.0* | -39 |  |  |  |  |  |

a: the first product ion (bold) was used for quantitation, and the second product ion (italics) was used for confirmation

b: the method detection limit (MDL) and method quantitation limit (MQL) were based on 10mg tissue samples
